# Mind the gap between non-activated (non-aggressive) and activated (aggressive) indoor fungal testing: impact of pre-sampling environmental settings on indoor air readings

**DOI:** 10.14324/111.444/ucloe.000055

**Published:** 2023-02-16

**Authors:** Spyros Efthymiopoulos, Yasemin D. Aktas, Hector Altamirano

**Affiliations:** 1 Department of Civil Environmental and Geomatic Engineering (CEGE), University College London, London, UK; 2 UK Centre for Moisture in Buildings (UKCMB), London, UK; 3 Institute of Environmental Design and Engineering (IEDE), UCL, London, UK

**Keywords:** mould, fungal testing, particles, indoor fungi, activated, non-activated, fungal growth assessment

## Abstract

Indoor fungal testing has been within the researchers’ scope of interest for more than a century. Various sampling and analysis techniques have been developed over the years, but no testing protocol has been yet standardised and widely accepted by the research and practitioner communities. The diversity in fungal taxa within buildings with varied biological properties and implications on the health and wellbeing of the occupants and the building fabric complicates the decision-making process for selecting an appropriate testing protocol. This study aims to present a critical review of non-activated and activated approaches to indoor testing, with an emphasis on the preparation of the indoor environment prior to sampling. The study demonstrates the differences in the outcomes of non-activated and activated testing through a set of laboratory experiments in idealised conditions and a case study. The findings suggest that larger particles are particularly sensitive to the sampling height and activation, and that non-activated protocols, despite dominating the current literature, can significantly underestimate the fungal biomass and species richness. Therefore, this paper calls for better-defined and activated protocols that can enhance robustness and reproducibility across the research domain focused on indoor fungal testing.

## Introduction

Indoor fungal growth may affect the health of occupants [1,2], disturb their comfort and well-being and lead to damage to the building fabric [3,4]. Therefore, it is of critical importance to be able to measure the extent of fungal growth correctly in a given indoor environment. Through testing, researchers aim to quantify fungal biomass, determine the conditions under which fungi flourish and assess whether the property needs remediation [5]. However, although many sampling and analysis techniques have been proposed and widely implemented [6], indoor fungal testing has not yet been fully standardised through well-established protocols [7,8].

**Figure 1:**
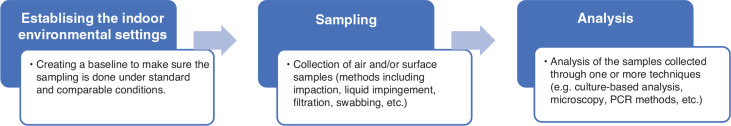
Schematic representation of the main stages of indoor fungal testing. (Source: Authors, 2022.)

Indoor fungal testing protocols are typically composed of the following steps: (1) establishing environmental settings prior to sampling, (2) collection of samples and (3) sample analysis via one or more techniques to estimate the amount and/or the contents of indoor fungal biota (Fig. 1).

The first stage of testing includes all the activities carried out prior to sampling, that is, the establishment of the indoor environmental settings. The preparation may be done by creating a completely still environment by restricting any access to, or movements in, the spaces to be tested for some time prior to testing (termed as non-activated sampling), or by resuspending particles through mechanical means such as an air blower for a predetermined amount of time, often defined as a function of the room surface area or volume (termed as activated sampling). Between these two extremes are the protocols where the equipment setup and some movement of the occupants or the investigators before the testing are allowed (Table 1).

**Table 1.** Some examples of non-activated (a, b) and activated protocols (c) (length of line segments not indicative of the durations associated with their labelled activities relative to each other)

Examples of different protocols	Example studies
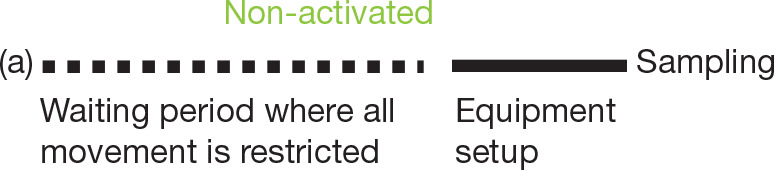	Terčelj et al. [9]: Windows and doors were closed for several hours prior to sampling Shinohara et al. [10]: Tests were carried out in unoccupied spaces
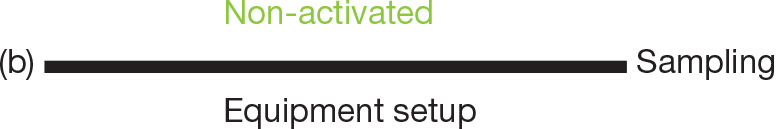	Gent et al. [11]; Dallongeville et al. [12]; Cahna et al. [13]; Nieto-Caballero et al. [14]: Activities before sampling were not restricted. The rooms were occupied before and during the sampling but no mechanical activation was carried out
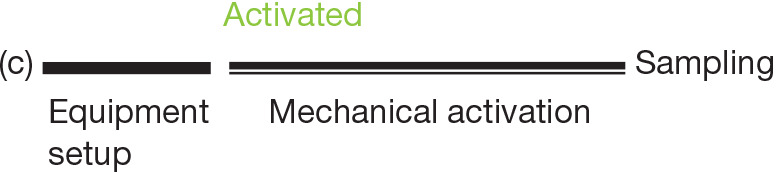	Aktas et al. [7,15,16]: Mechanical activation for approx. 1 min/10 m^2^ was carried out after the equipment setup. Upon completion of the activation, sampling was initiated

(Source: Authors, 2022.)

As seen, while there is substantial literature on the selection of sampling and analysis techniques depending on the aim of the investigation and the availability of tools [5], the standardisation of protocols with regard to the preparation of the indoor environment prior to sampling has gained limited attention and interest [17,18,19]. This situation manifests as additional complexity when comparing and contrasting the findings of multiple research studies, even when the same sampling and analysis techniques are used.

### Non-activated (non-aggressive) and activated (aggressive) protocols

The preparation of the environmental settings prior to sampling is often described by different and conflicting terms used interchangeably and without sufficiently clear definitions in the relevant literature, which leads to confusion. That is why a terminological discussion to supplement the work presented here was deemed necessary: the most common terms are active, activated and aggressive versus passive, non-activated and non-aggressive sampling to discriminate between resuspending the air, or not, before sampling [20]. Aktas et al. [7,15,16], Heinsohn [5] and Christensen and Swaebly [21] used the term *active* sampling to describe the collection of airborne particles after the disturbance of the air through artificial means, while *passive* sampling is used to describe the collection of particles from still air. Other researchers have used the same terms to differentiate between air sampling methods that require the creation of an artificial airflow for the collection of particles (e.g., impaction, liquid impingement, filtration and electrostatic precipitation) and methods that utilise gravitational settling (e.g., sedimentation), respectively [22–24]. To avoid common confusion over this overlap, in this paper we use the terms *activated* (*aggressive*) and *non-activated* (*non-aggressive*) to indicate still and perturbed indoor air conditions prior to sampling.

**Figure 2:**
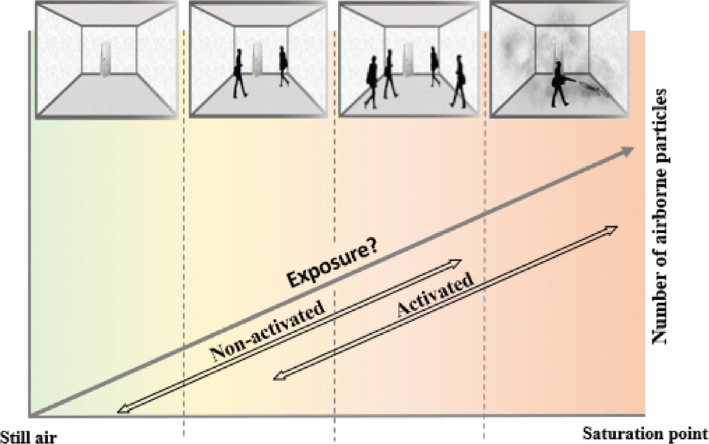
Illustration of non-activated (non-aggressive) and activated (aggressive) protocols. (Source: Authors, 2022.)

Terminological discourse aside, the boundary between what is considered to be still or actively mixed air is also often unclear, which has particularly dire implications for studies focussed on exposure (Fig. 2). Cahna et al. [11] and Nieto-Caballero et al. [14] collected air samples for the assessment of fungal contamination in classrooms while occupied by children. In another study by Shinohara et al. [10], house dust samples were collected from unoccupied houses in Japan, however there was no waiting time between equipment set-up and sampling. Aktas et al. [15] used a hand-held blower for 1 min/10 m^2^ to resuspend particles prior to sampling. In all three cases, the environmental settings under which the air sampling was carried out were different and the findings were not comparable. Although the use of a blower in the last example might be best placed to reach the ‘saturation point’ and can therefore be safely termed as an activated testing exercise (Fig. 2), where the other two cases sit within the wide spectrum of different levels of air stillness is unclear.

### Effect of activities and environmental context on the sampling readings

Many researchers have reported that the level and nature of the activities within a given indoor space do influence the extent and rate of resuspension of bioaerosols, including the fungal matter (Table 2). The creation of artificial air currents due to the disturbance of the air’s steadiness was found to increase the concentration of the airborne fungi through resuspension, hence allowing the detection of particles that are otherwise not detectable [15,26]. However, how the readings obtained from non-activated and activated protocols differ is yet to be fully quantified. Importantly, the relevant literature is dominated by the use of non-activated protocols [12,13,32,33]. This is primarily on the claim that the collection of samples from still air can reduce reproducibility-related errors [5], although several studies using both protocols comparatively suggest otherwise [7,15,28].

**Table 2.** Effect of the activation to the particle resuspension and fungal readings reported in the literature

Research by	Context/sampling and analysis method	Outcomes
Flannigan [25]	Fieldwork/impaction and colony enumeration	Suggested that during the occupied hours, the human activity led to a significant increase in the resuspension of fungal particles in the restaurant tested leading to an increase in the viable fungal counts (*approx. 16 times higher* than the readings during unoccupied hours)
Rylander [26]	Fieldwork/filtration and NAHA measurements	Reported that the indoor fungal levels (NAHA levels) in rooms in villas and apartments with no mechanical ventilation increased *up to 10 times* when an activated protocol using a blower was implemented compared to the readings when a non-activated protocol was selected
Aktas et al. [15]	Fieldwork/impaction on agar plates and colony enumeration and filtration and NAHA detection	Reported an *up to 56 times* increase of the activated fungal readings compared to non-activated readings when testing was carried out in residential buildings in North London
Mundt [27]	Laboratory experiments/particle counters used to measure airborne particle concentrations	Reported an *up to 10 times* increase in the concentration of PM5 and PM10 when a person entered, and vigorously walked around the test room
Mukai et al. [28]	Laboratory experiments/particle counters used to measure airborne particle concentrations	Reported an *up to 10 times* increase of the relative resuspension rate of particles ranging from 0.5 to 20 μm when the turbulence intensity and air velocity directly above three substrates (linoleum, metal and carpet) increased by 23% and 20 m/s, respectively
Goldasteh et al. [29]	Laboratory experiments/particle counters used to measure airborne particle concentrations	Reported 1) approximately *10 times* higher resuspension rates of 1–10 μm particle from hardwood flooring and 2) approximately *2–3 times* higher resuspension rate of 1–10 μm from linoleum flooring when airflow speed increased from 4.5 to 21 m/s in a laminar flow wind tunnel
Napoli et al. [30]	Fieldwork/impaction and total viable counts (TVC enumeration)	Reported an increase of the mean TVC by approximately *7.5 times* during operations in hospital rooms in the Apulia Region in South-eastern Italy
Wang et al. [31]	Fieldwork/particle counters used to measure airborne particle concentrations	Reported approximately *5.5 times* increase in average PM2.5 concentration when the occupancy density increased from 10 to 35 people in a classroom of Jinnan Campus of Nankai University

(Source: Authors, 2022.)

There are a number of parameters which would define the nature and extent of activation and would impact the readings in conjunction with it. The first and most immediate one is the changes in the ***pattern and the speed of the inspector’s movement across space*** which may lead to the resuspension of particles from different locations, potentially contaminated by fungi. Findings by Rylander [26] indicate that the movement of inspectors inside residential buildings prior to testing increased the measured concentration of fungal biota by two times compared to when all movement was strictly prohibited. Small-scale experiments and computational fluid dynamics (CFD) tests carried out by Cao et al. [34] indicated that human movement within a room could affect the dispersion of particles and that increase in the movement’s velocity is likely to lead to longer resuspension of fine particles (0.02–1 μm).

Another important parameter is the effect of the *room characteristics/condition*. Room characteristics, especially the level of cleanliness, can be connected to the amount of dust within a property and abiotic factors such as water and nutrient availability affecting the sporulation of certain fungal species [7,15,16,17]. In different properties, the quantity of dust and the fungi stored in it can vary. The volatility of fungi in dust can also change [35]. Hence, the particle aerosolisation rates may vary due to the different dust levels and volatility of fungal particles in every space and thus potential underestimation/overestimation issues regarding fungal readings may rise. Another important concern regarding the detectability of fungal particles, especially the larger ones, is how quickly the airborne fungal levels return to the pre-activation levels. Activities may lead to different levels of resuspension of particles of different sizes. Allowing a long time to pass after the end of the resuspension may lead to the settlement of heavy particles before they can be collected through sampling [36]. Mundt [27] suggests that large particles are expected to settle faster than small ones once indoor activities have stopped. To that end, the inability to specify the duration of time needed between the preparation of the indoor environment and the sampling might lead to variations in the sampling results.

Finally, another concern with regard to both the comparability of the testing outcomes from the available testing procedures and their ability to assess the appropriate potential fungal reservoirs is the uncertain impact of the *hygrothermal conditions* on the readings. The relative humidity affects the fungal growth and sporulation [37–39] directly but can also indirectly influence the resuspension rates and the recovery efficiency of fungal particles. Depending on the fungal species present the optimal relative humidity is likely to increase the spore production rate and conidial growth [37–39]. However, the moisture content in the indoor environment and the indoor materials can also affect the adhesion of fungi on the indoor aces and may vary across different spaces [40]. In high humidity conditions, liquid molecules can be adsorbed on small-sized bioaerosols, including small-sized (>0.1 μm) fungal particles leading to an increase in the adhesion forces between the particles and the surfaces they come in contact with [41]. As a result, the aerosolisation rate of particles by implementing the testing methods may vary from case to case, thus influencing the sampling readings.

We, therefore, raise the following open research question: *What environmental settings prior to sampling ensure that the testing procedure is replicable, and lead to comparable results from different properties?* This paper aims to fill this gap by detailing the impact of the indoor environmental settings prior to the sampling on the testing outcomes through a critical review of the literature (Materials and methods), a series of laboratory experiments and a case study demonstration (Results) to then discuss research and knowledge gaps, and implications on the practice (Discussion) and draw conclusions (Conclusions).

## Materials and methods

Experimental work and a case study testing demonstration were performed to examine the effect of the environmental settings on the resuspension rates of particles including fungal ones. Different protocols utilising air activation via the resuspension of particles with a leaf blower were implemented in both segments of the study. While the experimental work aimed to test the effect of different activated protocols on the concentration of airborne particles of different sizes in a controlled environment, the case study was designed to examine the applicability and validity of the experimental outcomes in real-life scenarios.

### Laboratory experiments

In order to investigate, on a quantitative basis, the impact of the level of ‘activation’ on the obtained readings, an experimental campaign was designed, and three sets of experiments were carried out. The experimental work aimed to identify the role of the blowing duration in the particle counts at different heights. The findings of this experimental work are then discussed in conjunction with the literature. For these experiments, particle counter readings were chosen as a proxy for indoor mould testing outcomes. This was considered a robust approach as fungal particles can range in size from 0.6 μm to 10 μm [42] and associations between the airborne fungal concentration changes and particle counts have been previously reported in the literature [43,44]. No cultivation and aerosolisation of fungal particles were carried out to minimise the risk of accidentally contaminating the test space.

**Figure 3:**
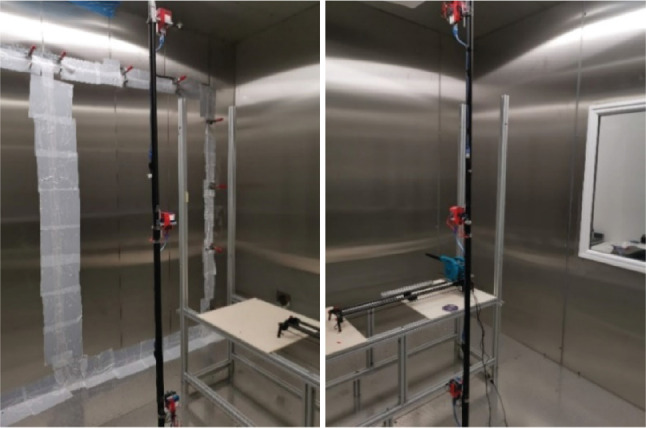
Experimental setup inside the environmental chamber. (Source: Authors, 2022.)

The work was conducted in an environmental chamber (2.8 × 3 × 3 m) where particle counters were positioned at three different heights (Fig. 3) – the readings from six particle size channels were analysed before and after activation. To ensure that dust or small particles cannot infiltrate the room through cracks and openings, the environmental chamber joints were entirely air-sealed with sealing tape typically used to perform blow-door tests. It is important to mention that the ventilation system in the chamber was not operated at any instance during these tests, and the ducts leading to the chamber were also sealed to avoid unintended disturbance in the air.

Three particle counters (PMS 5003 by Plantower [Nanchang City, Jiangxi Province, China], sensitivity: 50% – 0.3 μm, 98% – 0.5 μm and larger; resolution: 1 μg/m^3^; work temperature: operating temperature −10°C to 60°C, humidity (work): 0–99%) were placed at heights of 0.75 m, 1.5 m and 2.25 m on a vertical pole located in the centre of the room to monitor the variation of different sized particles with height. All three sensors were calibrated by the manufacturer prior to use.

**Figure 4:**
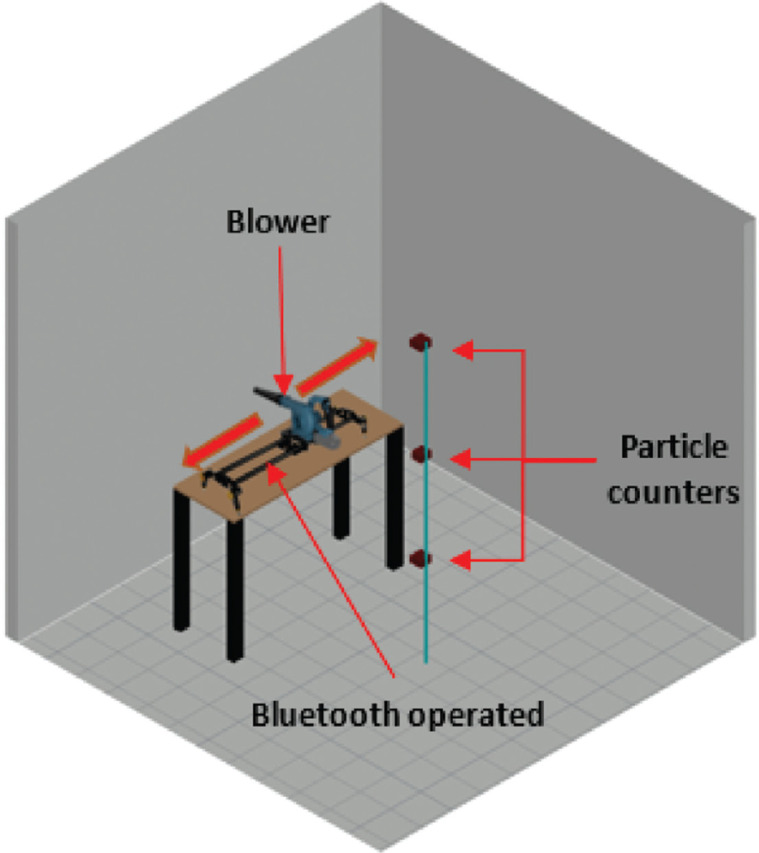
Schematic representation of the experimental setup and the movement of the blower inside the environmental chamber. (Source: Authors, 2022.)

To implement activated protocols within the environmental chamber, a leaf blower (Model 100760 Merry Tools’ [Katsu Tools, Harrow, UK] air leaf dust blower electric inflator) was used. The blower was placed on top of a Bluetooth-operated slider (Neewer’s [Shenzhen, China] motorised camera slider, maximum travel distance: 1 m) and was connected to a Wi-Fi-enabled plug to ensure that it could be operated remotely, without entering the chamber. The blower was set at a fixed height (distance between nozzle end and wall = 1.3 m) and a fixed distance from the wall (distance between nozzle end and wall = 1.5 m) and was able to move horizontally for a distance of 1 m (Fig. 4).

Experiments were conducted to examine the effect of six different blowing durations on the concentration of particles at the three different heights in the centre of the room. Tests were repeated three times (Series 1, 2, and 3 in Fig. 6) to reduce the likelihood of anomalous results and increase the accuracy. The blowing durations were set to be 1 min, 2 min, 3 min, 5 min, 10 min and 15 min. During these periods, the blower was set to move horizontally from one end of the slider to the other, while blowing towards the wall at a rate of 3.5 m^3^/min.

The experiments were conducted for a total of 20 days, and the chamber was closed and sealed until all experiments had been completed. Once the setup was complete, two days were allowed to pass before the experiments were initiated to ensure that all the resuspended dust due to the activities carried out for installation of the apparatus had settled down and different-sized particles’ concentration did not significantly change with time. The experiments were then initiated, and one experiment was carried out each day to ensure that the particles before the start of every experiment were in equilibrium. During these experiments, no dust was added inside the chamber, and the specific amount present inside the chamber was not known. However, knowing the exact amount of dust was not a prerequisite in this paper’s context, as this study aimed to identify how different activities would comparatively impact on the resuspension rates. Under no circumstance was anyone allowed to enter the room before the completion of all the experiments.

The particle counters were set up to start logging measurements 1 h before the start of the activation and continued until 8 h after it. The logging interval was 1 min and the logs represent the average number of particles counted every second.

### Experimental outcomes

**Figure 5:**
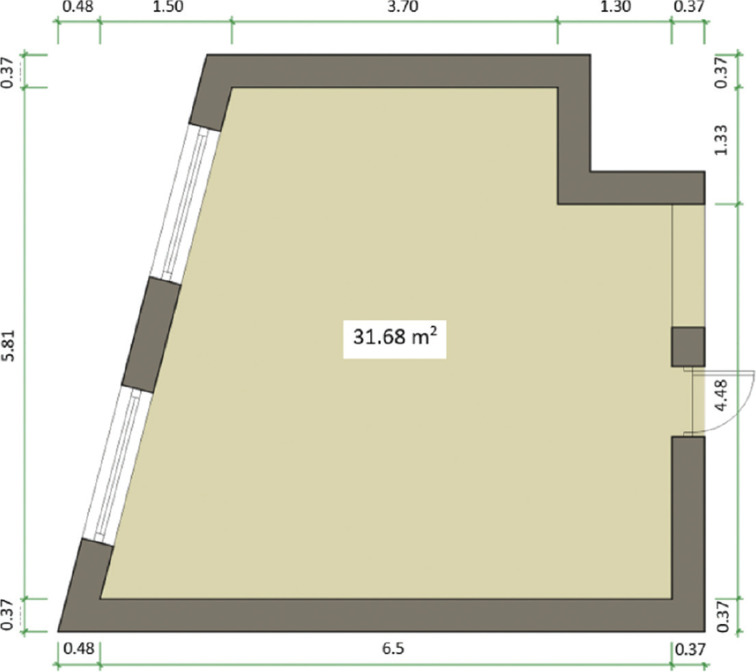
Floor plan of the case study living room (Source: Authors, 2022.)

To validate the experimental data and understand the effect of different non-activated/activated protocols to air sampling fungal readings in real case scenarios, six experiments (Table 3) were conducted early in September 2022 in the living room of an apartment in east London (Fig. 5). The property was located on the third floor of a high-rise residential building and no visual signs of mould or dampness were detected anywhere inside. The occupants allowed the performance of the tests on the property and did not enter the apartment for the whole duration of the experiments to avoid affecting the sampling readings in any way.

**Table 3.** Description of activities carried out in every test before sampling

Experiment	Description
***1***	No activity: The apartment remained unoccupied for more than 8 h to allow any resuspended particles to settle before the commencement of the air sampling. Before initiating the sampling, the covers of the filters were removed by pulling a string from outside the living room
***2***	Light activity: The investigator walked inside the room for 2 min without moving objects, furniture or wiping dust off from any surface
***3***	Medium activity: The investigator opened the window and walked inside the room for 5 min. Any other activity was restricted
***4, 5 & 6***	High activity: The investigator used a leaf blower (Makita BUB182) for approx. 1 min per 10 m^2^ (i.e., 3 min for the approx. 30 m^2^ case study room), to resuspend particles from any indoor surface (the distance between the investigator and the surface being blown was instructed to be 2 m)

(Source: Authors, 2022.)

The tests were designed to evaluate the effect of four different environmental settings on the sampling readings, which aimed to reflect different air-mixing (activation) states before sampling and involved carrying out what was deemed as no, light, medium and high-level activities (Table 3). The experiments for no, light and medium activity were performed once while the high activity case was repeated three times to investigate whether an activated protocol can be robust and reproducible.

The equipment was installed approximately 8.5 h before the commencement of the first experiment. Upon completion of the setup, the living room doors and windows were closed and permission to enter the room was restricted to allow resuspended particles to settle and avoid accidentally aerosolising any fungal particles from indoor surfaces. The first experiment was performed remotely using smart plugs to operate the pump and a DIY mechanism to uncover the inlet from the filters. However, while for the first experiment all activities were restricted for more than 8 h prior to sampling, the next five experiments were conducted consecutively with approximately 10 min between each and the investigator entered the space and performed the sampling manually. For all experiments the investigator wore an FFP3 mask for respiratory protection purposes.

Air sampling via filtration was used to measure indoor fungal biomass and species after different levels of activities, representative of different activated/non-activated protocols. Two mixed cellulose ester (MCE)-membrane filters (pore size 0.8 μm) provided by Mycometer A/S (Dr Neergaards Vej 3, 2970 Hørsholm, Denmark) were used for each experiment, to quantify the fungal biomass present in the sampled air. One polytetrafluoroethylene (PTFE) filter (pore size, 0.3 μm) preloaded in a 37-mm cassette was also used in every experiment to collect fungal DNA and detect the presence of 16 targeted species in the sampled air. The sampling time and volumetric flow rate were selected as 10 min and 15 l/min, respectively, for both types of filters. The filters were located within holes, cut based on the filter dimensions and at 75 cm from each other, through a 5-mm thick acrylic panel, fixed on top of a tripod such that the sampling is done at a height of around 1.3 m. This panel was built by the researcher in an effort to conduct both β-N-acetylhexosaminidase (NAHA) and DNA sampling simultaneously. An SKC BioLite+ (Blandford Forum, UK) with adjustable backpressure that allowed flow calibration was used for the DNA sampling, while a pump provided by Mycometer A/S was used for the NAHA testing. The tripod was located in the middle of the room for sampling. Upon completion of the tests, the MCE-membrane and PTFE filters were stored for 1 day at room temperature and in a −80°C freezer in-house and were sent to Mycometer A/S and HouseTest ApS (Petersmindevej 1A, 5000 Odense, Denmark) 1 and 2 days after the sampling, respectively.

The quantification of the fungal biomass was performed by Mycometer A/S using fluorogenic detection of different hydrolase activities (NAHA; EC 3.2.1.52). Hydrolase substrate containing a fluorophore was contacted with the membrane MCE filters. The enzyme activity was determined by measuring the amount of fluorescence formed during the reaction (following the protocol of the manufacturer, Mycometer A/S). The fluorescence signals from the fungi and the total allergens were then extracted and reported in relative fluorescence units (RFU), along with the Fungal to Allergen Index (FAI) (ratio of the fungi to allergen levels).

Species identification was performed by HouseTest ApS through the extraction and amplification of DNA from the PTFE filters. Quantitative polymerase chain reaction (qPCR) analysis was performed using primers targeting the DNA of (a) 16 fungal species (*Acremonium strictum*, *Alternaria alternata*, *A. fumigatus*, *A. versicolor*, *A. niger*, *Chaetomium globosum*, *C. cladosporioides*, *C. herbarum*, *C. sphaerospermum*, *P. chrysogenum*, *P. expansum*, *Rhizopus stolonifer*, *Stachybotrys chartarum*, *Trichoderma viride*, *Ulocladium chartarum*, *Wallemia sebi*), and (b) three fungal groups (*Aspergillus glaucus* grp., *Mucor/Rhizopus* grp., *Penicillium/Aspergillus/Paecilomyces variotii* grp.). The internal transcribed spacer (ITS) region of the nuclear ribosomal repeat unit was targeted by the primer sequences used as described by Lu et al. [45].

## Results

### Laboratory experiments

**Figure 6:**
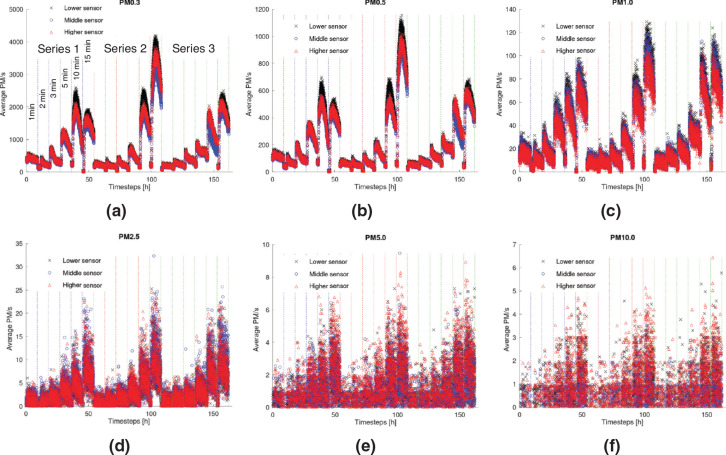
Particle counts for (a) PM0.3, (b) PM0.5, (c) PM1.0, (d) PM2.5, (e) PM5.0 and (f) PM10. Different segments in each plot delineated in different colours indicate three repeated sets of each test. The findings are indicated for 1, 2, 3, 5, 10 and 15 min of blowing and for each of the three series as shown in (a). (Source: Authors, 2022.)

The outcomes of the experimental work are shown in Fig. 6. The results indicate that the particle resuspension rates increase with the increase of the blowing time for all three sets of experiments, with up to X and Y times difference for small (particle matter [PM]a-b) and large particles (PMc-d), respectively, between sampling in still conditions and after 15 min of activation. Importantly, for blowing durations of more than 5 min, the smaller particles (particle size <PM1) did not return to the levels before the blowing was initiated, even after 7 h (Fig. 6a–c). However, the same cannot be stated for larger particles – for the same blowing duration, the pattern of the particle settlement becomes more unclear with the increase in the aerodynamic diameter (AD) (Fig. 6d–f).

**Figure 7:**
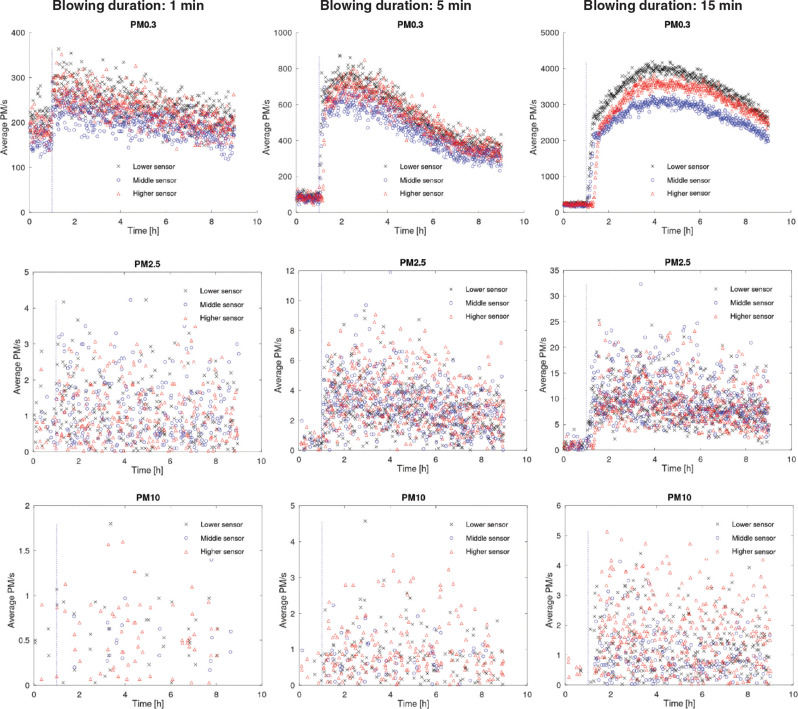
Measurements of PM0.3, PM2.5 and PM10 at different heights for 3 blowing durations (1 min, 5 min and 15 min). The blue vertical dashed line represents the commencement of the blowing. (Source: Authors, 2022.)

To better understand the pattern of the particle readings with the increase of the blowing duration, the average number of the PM0.3, PM2.5 and PM10 over 1-min intervals were plotted against time for three cases of blowing duration: 1 min, 5 min and 15 min (Fig. 7). As seen, higher blowing durations led to higher resuspension rates for all three size particles at any height.

**Figure 8:**
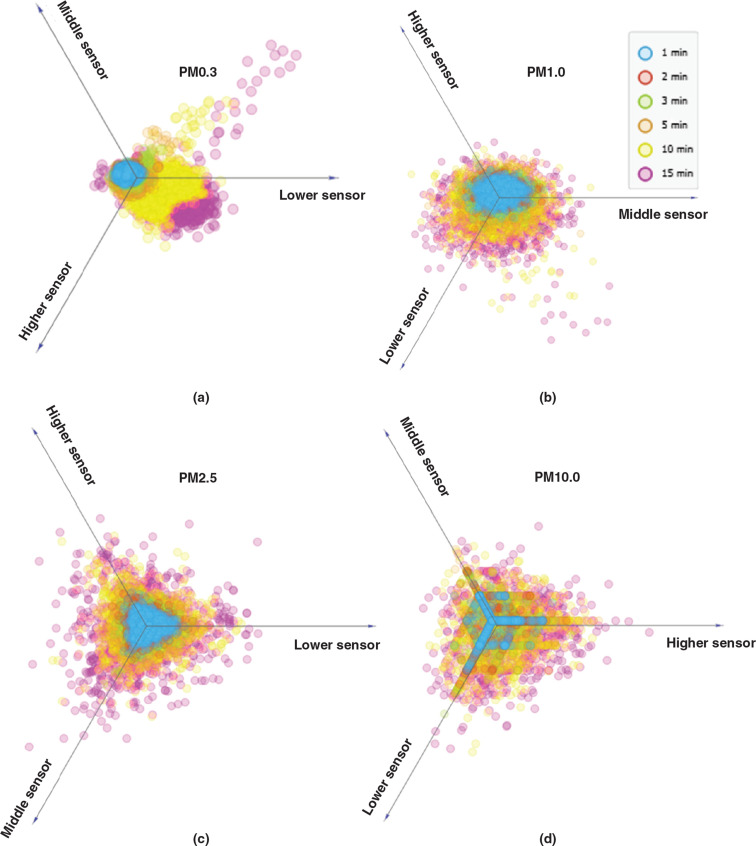
Linear projection of (a) PM0.3 (b) PM0.5 (c) PM2.5 and (d) PM10.0 at different heights. (Source: Authors, 2022.)

The number of different-sized particles measured at different heights was linearly projected to further study the effect of the blowing duration on the particle counts with height (Fig. 8). The increase in the blowing duration affected the resuspension rate of both small and large particles. The difference in the particle numbers due to the change in the blowing duration is better captured by the lower particle sensor than the middle and higher one (Fig. 8a). However, Fig. 8c–d shows a more uniform increase in the levels of the particles when their AD is larger than 1 μm with increasing blowing durations compared to the levels of PM0.3 and PM0.5 (Fig. 8a–b). Higher levels of the small particles (PM0.3 and PM0.5) were measured by the lower sensor than the higher ones.

**Figure 9:**
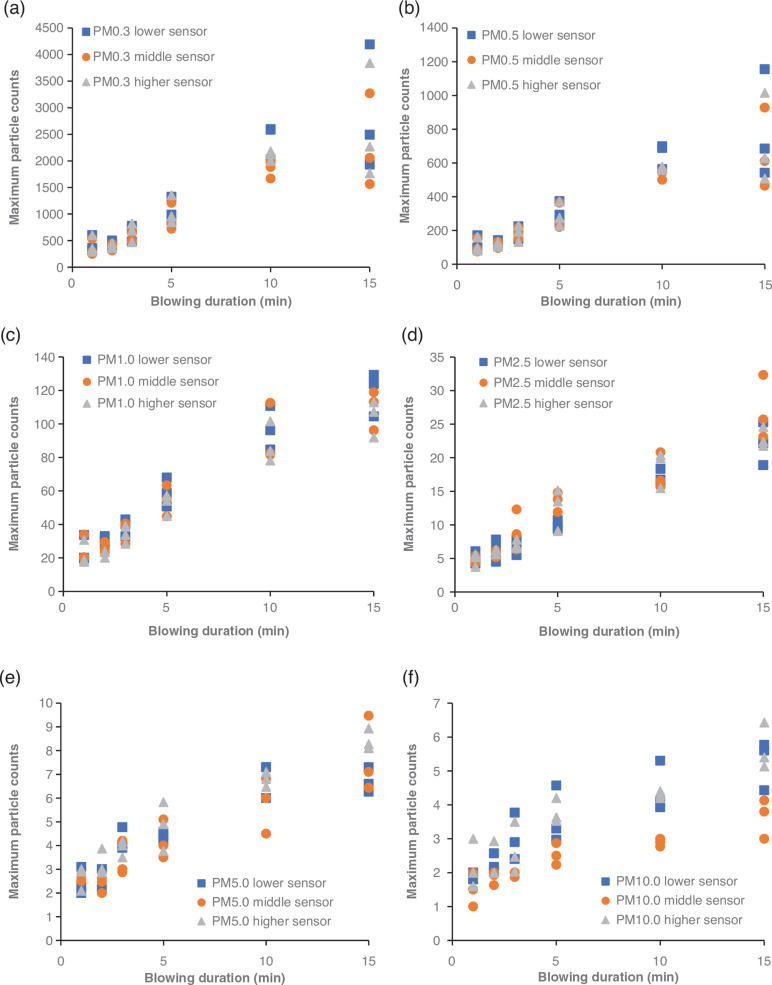
Maximum levels of (a) PM0.3 (b) PM0.5 (c) PM1.0 (d) PM2.5 (e) PM5.0 and (d) PM10.0 with height for the three experimental series. (Source: Authors, 2022.)

The maximum number of particles measured at each height in every experiment was plotted against the blowing duration and is demonstrated in Fig. 9a–f. The coefficients of determination [R^2^] for all three series suggest that the maximum number of particles of all sizes is strongly correlated to the blowing duration with the PM1.0 and PM2.5. In conjunction with Figs 6 and 7, the increasing trend of the maximum values of particles with the prolongation of the blowing duration suggests that the increase of the activity duration prior to sampling leads to higher particle resuspension rates.

Pearson’s correlation coefficient was calculated to identify potential correlations between the number of different-sized particles and the height, and a correlation matrix was created (Table 4). The results of the analysis indicate that the small particles (PM0.3–PM2.5) correlate strongly with each other regardless of the height of the sensor (r > 0.6). On the other hand, no strong correlations between the larger particles (PM5 and PM10) were identified with the highest value of r being 0.215 for PM5 particles measured by the higher and lower sensor and the lower R-value being the one for PM10 particles measured by the lower and higher sensor (r = 0.058). It should be noted that in a previous study by Luoma and Batterman [46], no significant variation was reported between readings of PM1 or smaller particles with height (particle counters placed at 0.4, 1.1, 1.8 m). On the other hand, the readings of particles ranging from PM5.0 to PM25 were shown to vary significantly with height [42].

**Table 4.** Correlation coefficient for same-sized particles measured at different heights

	PM0.3 low sensor		PM0.3 high sensor
PM0.3 low sensor	1.000		
PM0.3 mid sensor	0.993	1.000	
PM0.3 high sensor	0.986	0.986	1.000
	PM0.5 low sensor	PM0.5 mid sensor	PM0.5 high sensor
PM0.5 low sensor	1.000		
PM0.5 mid sensor	0.993	1.000	
PM0.5 high sensor	0.985	0.986	1.000
	PM1.0 low sensor	PM1.0 mid sensor	PM1.0 high sensor
PM1.0 low sensor	1.000		
PM1.0 mid sensor	0.964	1.000	
PM1.0 high sensor	0.951	0.953	1.000
	PM2.5 low sensor	PM2.5 mid sensor	PM2.5 high sensor
PM2.5 low sensor	1.000		
PM2.5 mid sensor	0.606	1.000	
PM2.5 high sensor	0.606	0.611	1.000
	PM5.0 low sensor	PM5.0 mid sensor	PM5.0 high sensor
PM5.0 low sensor	1.000		
PM5.0 mid sensor	0.142	1.000	
PM5.0 high sensor	0.215	0.211	1.000
	PM10 low sensor	PM10 mid sensor	PM10 high sensor
PM10 low sensor	1.000		
PM10 mid sensor	0.096	1.000	
PM10 high sensor	0.058	0.090	1.000

(Source: Authors, 2022.)

The Levene test was used to examine the equality of the variance for the number of same-sized particles measured by the same sensor during all three experiments. For a significance level of *P*_value_ = 0.05, the tests have indicated no homogeneity of the variances between the three experimental series for all cases tested. This suggests that although the tests were repeated in the exact same way the variances of the readings are significantly different. These differences might be a result of the limited horizontal movement of the blower and the limited area being directly affected by the high air velocity output. Nevertheless, this conforms with the literature outcomes in the Materials and methods section and adds extra value to the statement regarding the comparability concerns due to limited control over the activities and the testing conditions prior to sampling.

**Figure 10:**
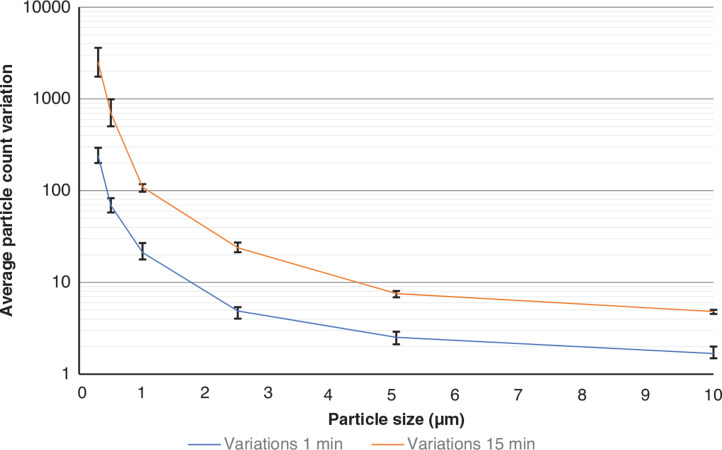
Minimum and maximum readings’ variations across the three series of testing for 1 min and 15 min blowing, respectively. (Source: Authors, 2022.)

Furthermore, to understand the extent of the activation’s effect on the particle readings, the differences between the maximum and minimum particle counts captured by every sensor were averaged for all experiments carried out for 1 min and 15 min blowing durations and plotted against particle size in Fig. 10. The blowing duration has led to an increase in the particle count variation for all particle sizes – with the difference being the largest for the smaller particles (>4000 particles for PM0.3 and 15 min blowing) and PM10 particles (<7 particles) and blowing duration of 15 min. This can indicate that the readings for the PM1.0 or smaller particles capture the blowing duration changes more easily than the larger particle counts. Therefore, monitoring the small-sized particles (<PM1.0) could be a better proxy of the intensity of the activities carried out prior to sampling. It is important to mention that despite the prolonged blowing duration (15 min), the variation in the large-particle readings (>PM2.5) was only slightly larger than the corresponding one when the blowing duration was 1 min, indicating that the air activation might be more critical for the recovery efficiency of PM2.5 and larger particles during the sampling.

### Case study outcomes

The Mycometer A/S method enabled the extraction of information with regard to three fungal growth indicators.

1. The fungal levels: Shows how many fungal particles have been detected in the first filter used during sampling.
2. The allergen levels: Shows the number of total allergens (dust mites, pollen, fungi, pet dander, skin cells, etc.) identified on the second filter used during sampling.
3. FAI: Measures the ratio between the two previous markers.

**Figure 11:**
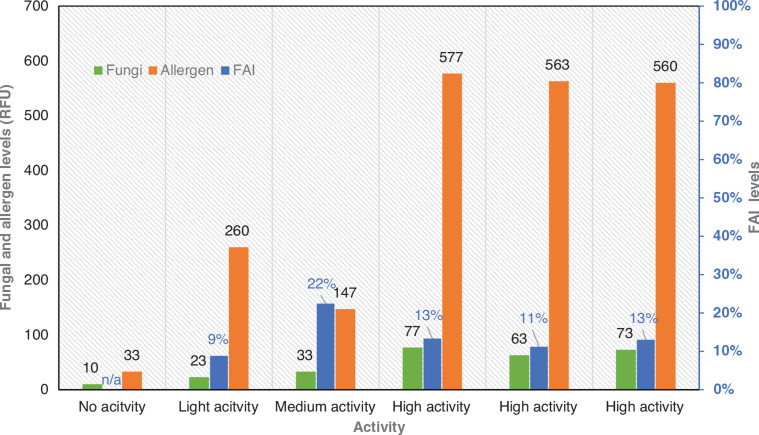
Fungal to Allergen Index (FAI), fungal and allergen levels measured from all experiments. (Source: Authors, 2022.)

The fungal and allergen levels measured in the case study room indicated that the intensity of the activities prior to sampling has a noticeable impact on the testing readings (Fig. 11). The first experiment (no activity) allowed the detection of very small concentrations of fungi (10 RFU) and allergens (33 RFU), while the results from the high activity experiments led to the capture of approximately seven and 17 times more fungal and allergen particles in the MCE filters, respectively. It should be underlined that although the fungal levels increased when the activities intensified, the allergen levels did not follow the same trend. The allergen readings from the medium activity experiment were found to be lower than the corresponding ones from the light activity experiment. The underestimation of the allergen levels in the tested space might have been a result of the opening of the windows and the air exchange between a potentially poorer-in-allergens outdoor environment and an indoor environment with higher allergen levels. It is also worth noticing that the FAI indicators did not follow a particular trend with the increase of the activities’ intensity. However, while the FAI ratios deviated noticeably in the first three experiments (no, light and medium activity), the deviation between the high activity ratios was rather minimal.

**Figure 12:**
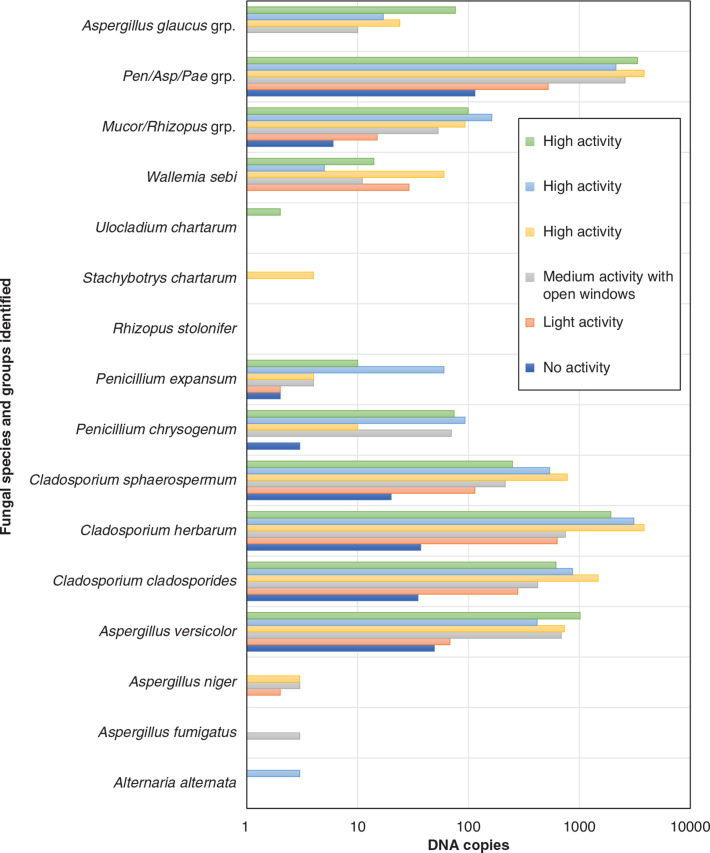
Fungal species identified from every case study test. (Source: Authors, 2022.)

Out of the 16 species that were targeted via the DNA amplification only six species were detected when activities prior to sampling were restricted completely (Fig. 12). However, with the increase of the activity’s intensity, the number of identified species rose to seven for light activity, 10 for medium activity, and nine, nine and 10 for high activity experiments. Although the number of DNA clones for every group or species identified varied with the increase of the activity intensity, the fact that the samples from the no and light activity tests have captured two to three species less than the medium and high activity tests might mean that non-activated protocols might be underestimating fungal activity in the rooms. In addition, *A. fumigatus* was only detected in a very small concentration in the sample from the medium activity test where the windows were open – this species that is known to live in compost and garbage outside [47] is likely to have appeared in the sample due to the air exchange between the indoor and outdoor environment. That *Uladicladium chartarum* and *Stachbotryn chartarum* appearing only under full activation conditions is also very telling, as they have larger spores than the others (around 8–12 × 4–5 μm and up to 20 × 16 μm, respectively).

## Discussion

The laboratory experiment has shown that the particle readings, regardless of the particle size, follow an increasing trend with the increase of the blowing duration. However, the impact of air activation on larger particle readings (>PM2.5) was considerably weaker than the effect of the smaller ones (<PM2.5). Previous studies [31,48] have shown that the indoor air velocity close to a dust reservoir and the activation method used can affect the particle resuspension rates. Still, they have also stressed that large particles might be more inert to activation due to their size and the strong adhesion bonds between them and the surfaces they are attached to. Therefore, investigators should consider that a strict non-activated protocol might seriously underestimate the particle intensity with important implications in relation to the indoor fungal levels and the health impact.

The particle counters’ low sensitivity for PM0.3 readings (50% sensitivity) indicates that the readings of this channel might not be an accurate proxy for the evaluation of the indoor activities before sampling. Still, they might be able to show a trend for the PM0.3 concentration. Given also that the majority of fungal particles will range between 1.0 μm AD and 3.2 μm AD [42], it can be stated that the PM0.3 readings would not be a suitable metric for the indoor fungal resuspension rates. On the other hand, the high sensitivity (>98%) of the sensor for particles larger than PM0.5 and the considerably higher particle count differences for smaller-sized particles (<PM1.0) with the increase of the blowing duration (Fig. 10) indicates that the PM0.5 and PM1.0 readings reported here could be the most appropriate indicators of the activities carried out before sampling.

The high correlation coefficients between readings obtained at different heights for <PM2.5 (Table 4) indicate that the height at which the sensors are placed will not significantly affect their ability to capture the concentration changes of particles. This conforms with previous studies showing that regardless of the sensor’s height, unlike the coarser particles (>PM5.0), the smaller airborne particle counts will follow similar trends due to their ability to spread throughout an indoor space unrestricted of their size [49]. However, the same cannot be stated for larger particles (>PM2.5) where the low correlation coefficients suggest that the height of the sensor had led to noticeable differences in the particle count changes with time.

When looking at the case study outcomes, the small deviations between the FAI, fungi and allergen levels from the high activity tests, especially when compared to the deviations between the other cases, suggest that activated protocols, if well-defined, are reproducible and provide robust fungal measurements. The results indicate that the increase of the activity intensity prior to sampling affects the fungal readings. The increase of the fungal readings with the escalation of the activities, comes in agreement with previous studies by Rylander [26] and Aktas et al. [15], who showed that the use of a blower prior to sampling led to an up to 10 and 56 times increase in the testing outcomes compared to the no and low activity readings, respectively. Airing the room before the sampling, such as through opening the windows, might lead to measurements that may not be representative of the indoor fungal activity due to the mixing of indoor and outdoor air, as shown by the medium activity testing in our case study.

The intensification of activities prior to sampling has allowed the detection of more species than the ones captured from the no activity case. However, while the low activity experiment allowed the identification of an additional species in the sample all other experiments led to the detection of three or more species. This is in broad agreement with the hypothesis that air activation can allow the aerosolisation and detection of fungal particles that develop stronger bonding forces with the substrate materials and would not be identified via non-activated protocols carried out prior to sampling. It is also noteworthy that species with large spores were not captured by tests done following a no-, low- or medium-level of activity – an observation in line with the experimental outcome that the large-size particles are harder to suspend and hence to sample.

## Conclusions

Non-activated protocols, currently dominating the literature, can heavily underestimate the fungal biomass and the species present within a given indoor space, with serious implications for studies focussed on health and building condition alike. Researchers should give attention to the conditions under which indoor fungal testing is carried out. Outcomes of non-activated and activated testing do differ, therefore indoor fungal levels cannot be evaluated or benchmarked unless uniformity is brought to the pre-sampling conditions through a well-established testing protocol. Disturbing the air’s stillness through activation does increase the concentration of captured airborne fungal fragments and spores, and thus leads to the detection of fungi that could otherwise be undetectable. The experimental work carried out here suggests that the use of a blower and the increase of the blowing duration lead to higher particle resuspension. In real-case scenarios this manifests as higher detectability of particles ranging from 0.3 to 10 μm, as supported also by the case study testing, we present here. The findings suggest that the smaller particle (PM0.3 to PM1.0) readings are more responsive to the different levels of activity than the larger particles (PM2.5 and PM5.0). The sensitivity of the testing outcomes to the level of activity within the testing space prior to sampling also suggests that the non-activated protocols, if chosen, must control very closely the use of the room for several hours prior to the sampling. In practice this may be very difficult, but an inability to control the conditions prior to sampling can lead to outcomes that are not comparable with other studies.

Therefore, the selection of an *activated protocol is of critical importance* for collection efficiency, both in terms of fungal biomass and the number of identified species. As shown by the repeated testing in our case study, a blowing duration of 1 min/10 m^2^ proves to be efficient to capture the fungal activity and leads to stable readings. Furthermore, the experimental study presented here suggests that the sampling height is another testing protocol variable that the larger particles are more sensitive to and should be paid attention to for comparability purposes. Our work shows that while any height between 0.75 and 1.5 m should work well, sampling at higher elevations might not be able to capture larger size particles. Furthermore, activities that could lead to an increase/decrease of contaminants indoors, such as the opening of windows, can have unclear effects on the fungal measurements and should be avoided.

## Data Availability

The datasets generated during and/or analysed during the current study are available from the corresponding author on reasonable request.
